# Mechanisms underlying the weight loss effects of RYGB and SG: similar, yet different

**DOI:** 10.1007/s40618-018-0892-2

**Published:** 2018-05-05

**Authors:** A. Pucci, R. L. Batterham

**Affiliations:** 1 Centre for Obesity Research, Rayne Institute, University College London, London, UK; 2 Centre for Weight Management and Metabolic Surgery, University College London Hospital Bariatric, London, UK; 3 National Institute of Health Research, University College London Hospital Biomedical Research Centre, London, UK

**Keywords:** Roux-en-Y gastric bypass, Sleeve gastrectomy, Gut hormones, Bile acids, Gut microbiota, Type 2 diabetes

## Abstract

The worldwide obesity epidemic continues unabated, adversely impacting upon global health and economies. People with severe obesity suffer the greatest adverse health consequences with reduced life expectancy. Currently, bariatric surgery is the most effective treatment for people with severe obesity, resulting in marked sustained weight loss, improved obesity-associated comorbidities and reduced mortality. Sleeve gastrectomy (SG) and Roux-en-Y gastric bypass (RYGB), the most common bariatric procedures undertaken globally, engender weight loss and metabolic improvements by mechanisms other than restriction and malabsorption. It is now clear that a plethora of gastrointestinal (GI) tract-derived signals plays a critical role in energy and glucose regulation. SG and RYGB, which alter GI anatomy and nutrient flow, impact upon these GI signals ultimately leading to weight loss and metabolic improvements. However, whilst highly effective overall, at individual level, post-operative outcomes are highly variable, with a proportion of patients experiencing poor long-term weight loss outcome and gaining little health benefit. RYGB and SG are markedly different anatomically and thus differentially impact upon GI signalling and bodyweight regulation. Here, we review the mechanisms proposed to cause weight loss following RYGB and SG. We highlight similarities and differences between these two procedures with a focus on gut hormones, bile acids and gut microbiota. A greater understanding of these procedure-related mechanisms will allow surgical procedure choice to be tailored to the individual to maximise post-surgery health outcomes and will facilitate the discovery of non-surgical treatments for people with obesity.

## Introduction

The prevalence of obesity continues to increase unabated. Globally in 2014, approximately 52% of the adult population were overweight (1.9 billion) or obese (> 600 million) [1]. Obesity increases mortality and its associated comorbidities including cardiovascular disease, type 2 diabetes (T2D) and some cancers, and represents a major health and economic burden.

Bariatric surgery is recognised as the most effective treatment for people with severe obesity, defined by a body mass index (BMI) equal to or greater than 40 kg/m^2^, or greater than 35 kg/m^2^ in the presence of obesity-related complications [2]. Bariatric surgery involves surgical modifications of the gastrointestinal (GI) tract anatomy with a consequent alteration of nutrient flow affecting GI biology [3]. Many clinical trials have demonstrated the superiority of bariatric surgery in terms of efficacy and sustainability of weight loss and resolution of obesity-related comorbidities when compared with intensive medical and lifestyle interventions [4–6].

The concept of bariatric surgery emerged during the 1950s when procedures that involved small intestine resection were noted to result in weight loss [7–9]. The first bariatric procedures were, therefore, designed to specifically induce weight loss through pure malabsorption somewhat predictably these procedures led to severe nutritional deficiencies and metabolic consequences. From the observation that patients undergoing gastric resection and/or bypass for peptic ulcer disease tended to lose weight, Mason and colleagues performed the first gastric bypass procedure in 1967 [10, 11]. This procedure combined reduced stomach capacity (restriction) and decreased digestion forming the basis for subsequent “malabsorptive” and “restrictive” procedures [10]. However, 50 years on, it is now accepted that most bariatric procedures engender weight loss and metabolic improvements by mechanisms other than restriction and/or malabsorption.

**Fig. 1:**
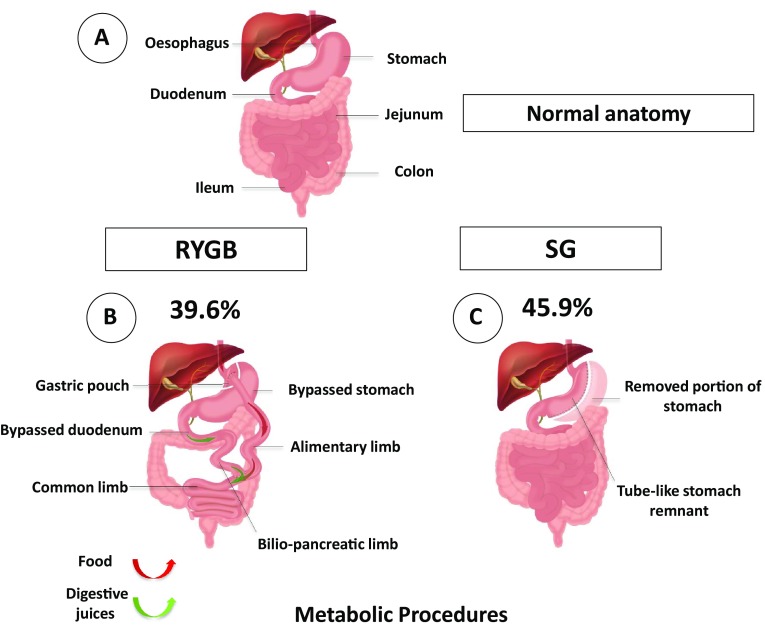
Schematic diagram illustrating the normal upper GI anatomy (**a**) and the two most commonly performed bariatric surgical procedures in the world with relative percentages. The metabolic procedures: **b** RYGB and **c** SG (surgical technique described in details in the main text). RYGB were the 39.6% and SG were the 45.9% of the total procedures performed in 2014 [12]. *RYGB* Roux-en-Y gastric bypass, *SG* Sleeve gastrectomy

Over the past decade, the effectiveness of bariatric surgery has resulted in a marked increase in the number of procedures undertaken worldwide, with approximately 580,000 operations performed in 2014 [12]. The surgical procedures undertaken are continuously evolving, based on technical advances, efficacy data, short-term and long-term complication rates, and increased understanding of the physiology underpinning their success. Currently, the most common procedures undertaken globally are sleeve gastrectomy (SG) (45.9%) and Roux-en-Y gastric bypass (RYGB) (39.6%), and these two procedures form the focus of this review. Purely restrictive procedures, such as adjustable gastric banding, are now less commonly performed (7.4%) [12] (Fig. 1).

Despite the increasing use of bariatric surgeries, the underlying mechanisms remain incompletely understood. Post-operative weight loss is highly variable [13] and many studies suggest that the total amount of weight loss plays a major role in determining glycaemic improvements and remission of comorbidities after surgery [14]. Given the difficulty of accessing bariatric surgery in many countries, it is crucial to identify patients who may benefit the most from surgery and to tailor the surgical procedure to the individual patient to maximise health outcomes. To achieve this aim, we need to gain a greater understanding of the mechanisms underlying the sustained weight loss produced by bariatric surgery, procedure-related differences and how these interact with the patient’s biology. This review provides an overview of mechanisms, suggested to contribute to weight loss after RYGB and SG.

## Roux-en-Y gastric bypass

In RYGB, the stomach is divided generating a small gastric pouch (20–30 mL), which is then anastomosed with the mid-jejunum, creating the Roux or alimentary limb. Ingested nutrients thus bypass most of the stomach, duodenum, and the proximal jejunum. Anastomosis of the biliopancreatic limb with the jejunum allows drainage of bile acids (BA) and pancreatic secretions, which mix with the nutrients in the jejunum (common limb) [15] (Fig. 1). A technically easier version of the standard RYGB, the one anastomosis gastric bypass (OAGB) is gaining favour (approximately 10,400 procedures worldwide in 2014, 1.8% of all bariatric procedures) but mechanistic studies and long-term outcome studies are awaited and OAGB will not be part of this review [12].

## Sleeve gastrectomy

Sleeve gastrectomy (SG), was initially performed as a first-stage procedure to reduce weight in patients with a BMI of greater than 50 kg/m^2^ and was intended as a purely restrictive procedure. However, the significant sustained weight loss and metabolic benefits obtained by SG led to its adoption as a standalone procedure. SG involves transection along the greater curvature creating a tube-like new stomach removing the fundus and body [16] (Fig. 1). Gastric contents pass rapidly into the duodenum. SG has become the most common bariatric procedure because of the easier technique, shorter operation time, fewer surgical and nutritional complications, and similar short-term weight loss and clinical outcomes compared with RYGB [12].

## Weight loss and metabolic benefits after RYGB and SG

It has been clearly demonstrated that bariatric surgery is an effective treatment for severe obesity engendering marked weight loss, sustained in the long term when compared to calorie-restricted dieting. The 20-year outcome data from the Swedish Obese Subjects (SOS) study showed that patients who received bariatric surgery achieved a significantly greater mean body weight reduction of approximately 18% compared with approximately 1% in patients who received standard medical treatment through their local health centres [5]. RYGB patients were able to maintain more than 25% of their total weight loss after 20 years (SG was not performed yet when the study started) [5]. Multiple retrospective uncontrolled observational studies and also randomised clinical trials (RCTs) have demonstrated the superiority of bariatric surgery both in terms of weight loss outcomes and resolution of comorbidities when compared with intensive medical and lifestyle interventions [4, 17]. The 5-year results from the STAMPEDE RCT, which recruited patients with obesity and T2D, clearly showed how bariatric surgery was more effective than intensive medical therapy in inducing weight loss and in decreasing, or even resolving, hyperglycemia [4].

A limited number of RCTs have compared the efficacy of RYGB against SG with regards to weight loss outcomes and resolution of obesity-related comorbidities, especially T2D. The STAMPEDE trial, which was not powered to detect differences between procedures, reported that RYGB was associated with greater weight loss and a need for fewer T2D medications after 5 years compared with SG. This is interesting considering that 3-year results from the same authors and from other short-term studies showed similar results for the two procedures [17–19]. The SM-BOSS RCT reported no significant weight loss difference between the two procedures at 5 years post-surgery [20]. The 5-year results from the SLEEVEPASS RCT showed how, although not statistically significant, RYGB was associated with greater weight loss, T2D remission, discontinuation of medications for dyslipidaemia and hypertension, the latter reaching statistical significance [21]. The 5-year results from the Strasbourg RCT confirmed this trend showing that RYGB resulted in more stable weight loss when compared to SG [22]. Furthermore, a recent meta-analysis including 15 RCTs reported that RYGB may provide a greater degree of weight loss at 2–5 years post-operatively compared with SG [23]. Interestingly, the difference in weight loss between RYGB and SG groups increased with time. Another small 2-year RCT reported that despite similar weight loss results, RYGB reduced truncal fat compared to SG. This differential impact upon truncal fat might in part explain why RYGB leads to greater glycaemic improvement than SG despite similar weight loss. However, larger studies are needed to investigate this finding [24].

## The physiology of body weight regulation

**Fig. 2:**
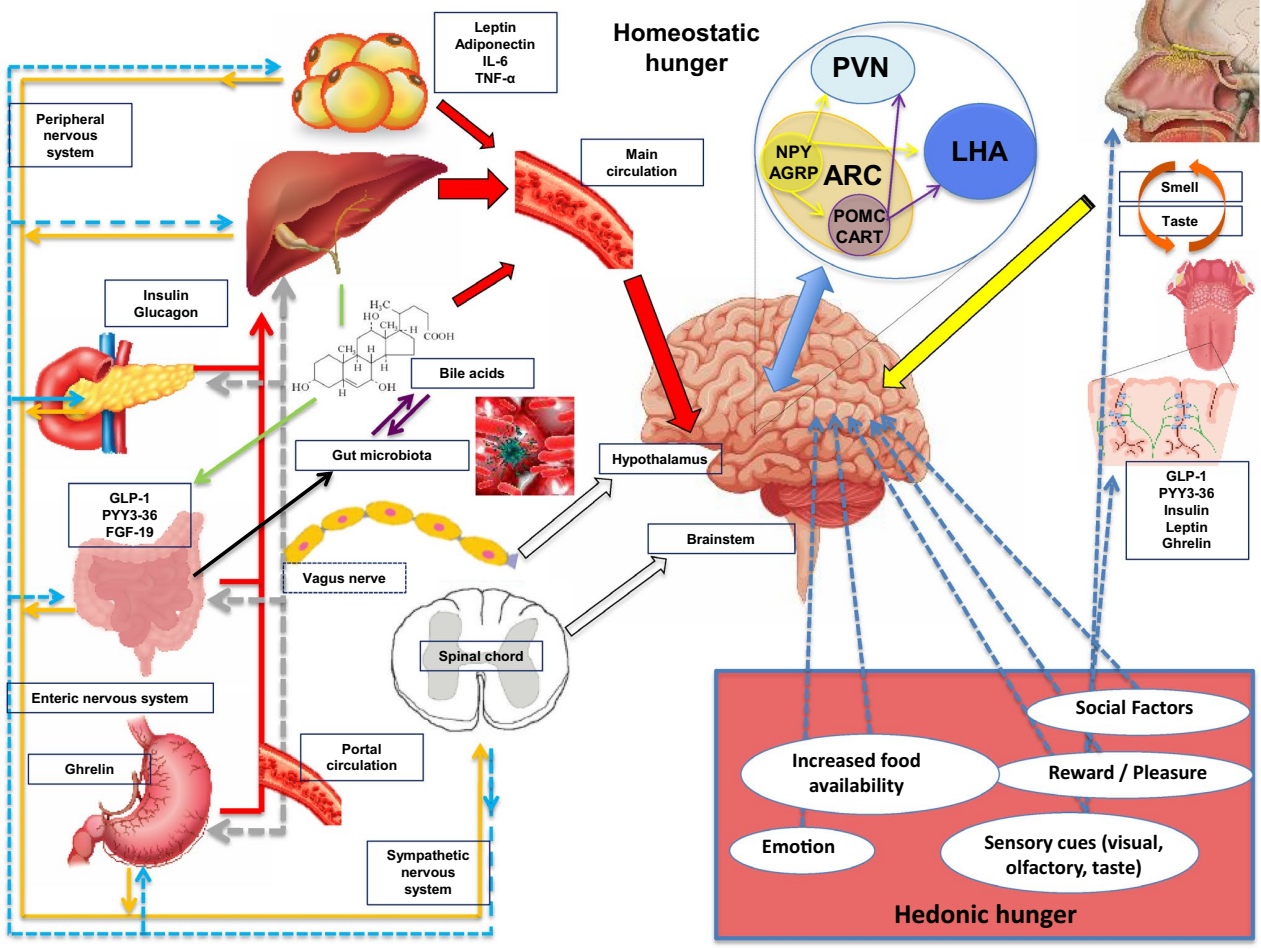
Schematic diagram illustrating the mechanisms involved in regulating feeding behaviour. Nutrient entry into the GI tract causes stomach and intestine distension, secretion of pancreatic enzymes and BA, altered enteric and vagal nerve signalling and exposure of gut enteroendocrine cells to nutrients with altered circulating gut hormone levels (e.g. decrease in orexigenic hormone ghrelin and increase in anorectic hormones PYY3-36 and GLP-1). Gut-derived signals (nutrients, hormones, and neural) and adipokines (e.g. leptin, IL-6, TNF-alpha and adiponectin) act directly and indirectly upon brainstem and hypothalamic arcuate nuclei (first order neurons: orexigenic NPY/AgRP and anorexigenic POMC/CART). ARC neurons interact with second order neurons in the PVN, and to the LHA. All those mechanisms are involved in the regulation of homeostatic hunger. Social factors, emotion, reward, pleasure, increased food availability and sensory cues can influence brain reward and higher cognitive brain regions leading to altered feeding behaviour (hedonic hunger). Taste and olfactory signals can also influence energy intake acting on both homeostatic and brain reward systems. Insulin leptin, GLP-1, PYY and ghrelin are present in saliva with cognate receptors on taste buds and olfactory neurons. *AgRP* agouti-related peptide, *ARC* arcuate nucleus, *CART* cocaine and amphetamine-regulated transcript, *FGF-19* fibroblast growth factor-19, *GLP-1* glucagon-like peptide 1, *IL-6* interleukin-6, *LHA* lateral hypothalamic area, *NPY* neuropeptide Y, *PNS* peripheral nervous system, *PVN* paraventricular nucleus, *PYY3-36* peptide tyrosine–tyrosine 3-36, *POMC* pro-opiomelanocortin, *SNS* sympathetic nervous system

Feeding behaviour is determined by homeostatic and reward-related brain centres that continually integrate peripheral signals relating to energy stores and nutrient availability [25] (Fig. 2). Obesity results when energy intake chronically exceeds energy expenditure, which in turn may be due to an alteration of the homeostatic or hedonic system or both [26]. Peripheral energy signals are classified as long term, such as leptin and insulin, which provide information regarding energy stores and short term, including nutrient and meal-derived energy availability messages [25]. Gut hormones are secreted from the GI enteroendocrine cells in response to nutrient ingestion and act as regulators of energy balance and glucose homeostasis [27]. The gut hormones, peptide YY3-36 (PYY) and glucagon-like peptide-1 (GLP-1), are secreted from enteroendocrine L cells present throughout the GI tract, in response to nutrient ingestion [27, 28]. Both PYY and GLP-1 have an appetite-suppressing effects, modulating neural activity within homeostatic and reward brain regions [28–30]. In addition, both PYY and GLP-1 impact upon glycaemic regulation [31]. GLP-1 is one of the key mediators of the incretin effect (the augmentation of insulin secretion after oral as opposed to intravenous administration of glucose) [29]. Furthermore, GLP-1-based medications are used to treat people with T2D and more recently obesity [32].

In contrast to the anorectic actions of PYY and GLP-1, ghrelin, produced primarily by P/D1 cells in oxyntic glands in the gastric fundus, stimulates appetite and energy intake. Circulating ghrelin levels increase in the fasted state and decrease post-prandially proportionally to the amount of ingested food [33]. Ghrelin also acts on homeostatic and reward centres, and elevations of ghrelin levels can enhance the hedonic responses to eating [34].

BA are produced in the liver, stored in the gallbladder and secreted into the duodenum upon nutrient ingestion. Their main role is the facilitation of micelle formation promoting the digestion of dietary fat and fat-soluble vitamins. More recently, BA have also been shown to play a role in regulating glucose and energy homeostasis [35]. BA activate GLP-1 secretion via activating G protein‐coupled receptors (TGR5) on L cells and fasting total circulating BA levels are positively correlated with postprandial GLP-1 levels [36]. BA also act on farnesoid X receptor (FXR) present in pancreatic β cells increasing insulin release [37]. BA activation of intestinal FXR cells stimulates the secretion of fibroblast growth factor-19 (FGF-19), a protein that contributes to improved peripheral glucose disposal and lipid homeostasis leading to reduced weight and increased metabolic rate [38, 39]. In animal studies, BA supplementation has been shown to reduce weight gain, [40] and postprandial BA levels are inversely related with body fat mass [41]. Thus, the physiologic effects of BA likely extend beyond the gut and pancreas with TGR5 receptors also located on skeletal muscle.

The human gut hosts trillions of microorganisms [42]. Gut microbiota can affect energy absorption, through altering intestinal mucosal permeability, energy expenditure by intracellular thyroid hormone activation via FXR signalling [40] and immunologic systems of their human hosts [43]. Diet, antibiotic exposure and other environmental factors can in turn affect the diversity of the microbiota and their function [43].

**Fig. 3:**
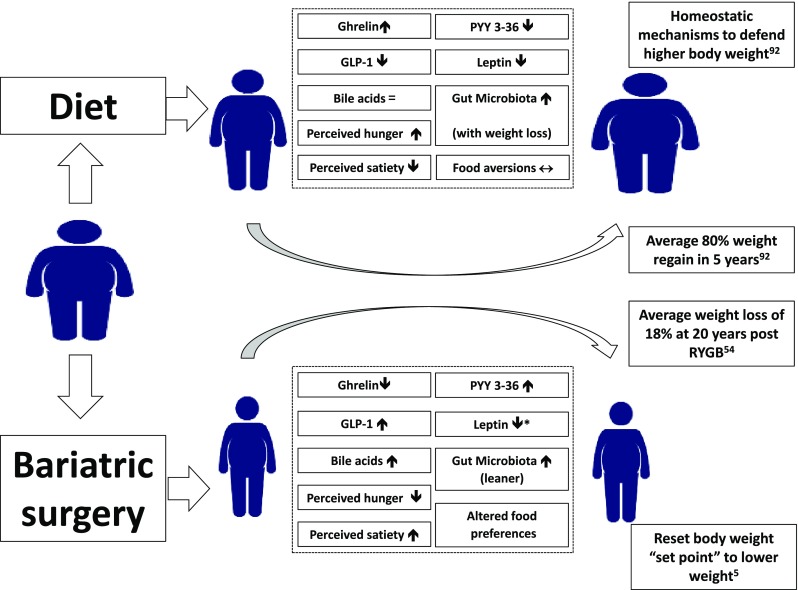
Schematic diagram illustrating the different biological changes induced by weight loss obtained through dieting (upper part) compared to bariatric/metabolic surgery (lower part). Powerful compensatory biological changes contribute to the high rate of weight recidivism observed following lifestyle intervention weight management. Many homeostatic mechanisms act to defend higher body weight, and these includes hormonal alterations and a decreased energy expenditure leading to increased hunger and energy consumption. In contrast, bariatric surgery leads to a favourable biology that includes increased satiety hormones, reduced ghrelin, enhanced BA secretion and a “lean” microbiota. Together these mechanisms lead to reduced hunger and a shift towards healthier food options with a resetting of body weight “set point” to a lower level facilitating meaningful and sustained weight loss. *GLP-1* glucagon-like peptide 1, *PYY3-36* peptide tyrosine–tyrosine 3-36. *Suggestion that leptin sensitivity may improve References for this figure [5, 60, 96]

Taste and olfactory signals can impact on energy intake by influencing food selection [44]. There is a close interaction between signals of energy homeostasis, and taste and smell. Insulin leptin, GLP-1, PYY and ghrelin have been found in saliva and their cognate receptors identified on taste buds and olfactory neurons [44]. Rewarding food-related sensory stimuli can override satiety signals leading to excess energy intake. The latter leads to deregulation of the homeostatic mechanisms that normally control body weight predisposing individuals to gain more weight [34] (Fig. 3).

## The obese state: pathophysiologic changes

Obesity is the result of a chronic positive energy balance [45]. Once the obese state is fully established, many pathophysiologic changes occur including leptin and insulin resistance together with reduced circulating plasma PYY and GLP-1 levels in response to nutrient ingestion. The postprandial suppression of circulating ghrelin is also reduced. Obesity has also been shown to blunt the rise in circulating postprandial BA levels [46].

A dysbiotic relationship between host and microbiome has been suggested to contribute to the development of obesity [47], with profound differences found between the microbiome composition of obese and lean individuals [48]. Obesity is associated with the relative increase or reduction of certain bacterial species and the importance of the relative proportions of those species remains an area of active investigation. Transplantation of gut bacteria from obese mice to normal weight germ-free mice results in weight gain in the recipients [49]. Conversely, faecal transplantation from lean human donors to recipient patients with metabolic syndrome led to improvements in insulin sensitivity. A dysbiotic relationship may affect host energy and nutrient metabolism altering intestinal mucosal permeability, promoting increased fat storage in adipose tissue [50], by enhancing the absorption of short-chain fatty acids derived by otherwise indigestible luminal polysaccharides and by triggering inflammatory responses through a process referred to as “metabolic endotoxemia” [51, 52].

The neural response to food cues is altered in people with obesity compared to people with normal weight. This has been confirmed by brain-imaging studies showing an increased stimulation of central reward pathways in response to eating or food cues [26]. In addition, there is evidence that eating behaviour in people with obesity becomes dissociated from perceptions of satiety and hunger [53, 54].

## Biological changes that favour weight recidivism following lifestyle interventions

Lifestyle interventions lead to weight loss. However, people with overweight and obesity find it very hard to maintain this weight loss in the long term. In response to weight loss, which throughout evolution would have been a threat to survival, multiple powerful biological changes occur that lead to increased hunger, enhanced neural responses to food cues and heightened drive to consume energy-dense foods. Compensatory changes include decreased energy expenditure, due to reduced lean muscle mass and reduced sympathetic activity [55], reduced circulating leptin, GLP-1 and PYY levels with increased ghrelin levels [54], and altered brain neural response to food cues. Impaired circulating BA levels, an altered gut microbiome, and decreased vagal signal transmission are also described [56]. These changes are summarised in Fig. 3 and contribute to the high rate of weight recidivism observed following lifestyle intervention weight management programmes [57].

## Biological changes that favour sustained weight loss following SG and RYGB

**Fig. 4:**
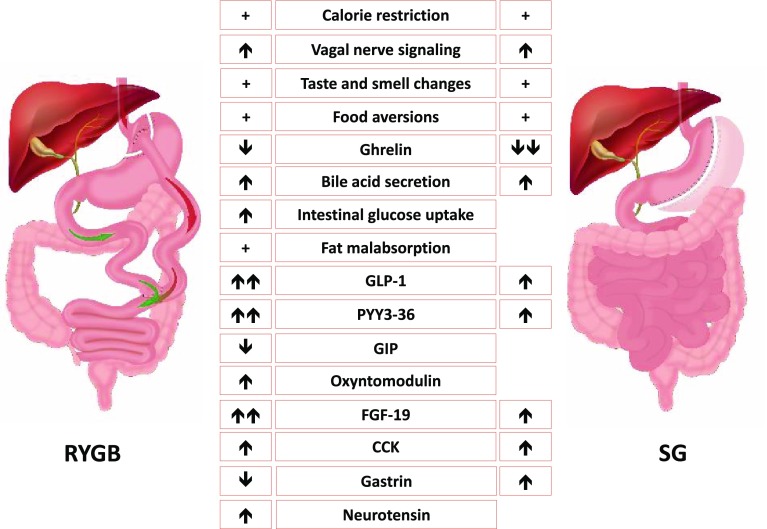
Schematic diagram illustrating RYGB and SG and the mechanisms leading to weight loss and resolution of comorbidities. For every mechanism the effect of the procedure is represented with a “↑” when stimulating or “↓” when suppressing. A “+” means that the proposed mechanism is present only after surgery when compared to the pre-operative period. When the effect is stronger for one of the two procedures there is a double arrow compared with a single one. When the effect is missing for one procedure it means that the mechanism is procedure specific. *RYGB* Roux-en-Y gastric bypass, *SG* Sleeve gastrectomy, *GLP-1* glucagon-like peptide 1, *PYY3-36* peptide tyrosine–tyrosine 3-36, *GIP* gastric inhibitory polypeptide, *FGF-19* fibroblast growth factor-19, *CCK* cholecystokinin

The multifactorial mechanisms promoting weight loss following RYGB/SG remain incompletely understood. However, it is clear that the beneficial effects are not achieved through malabsorption and restriction alone [58, 59]. Reduced energy intake, as a result of altered eating behaviour, is recognised as the main driver for weight loss. In contrast to the compensatory biological changes that are seen following weight loss induced by lifestyle interventions, SG and RYGB are associated with reduced hunger and reduced neural responsiveness to food cues. Moreover, food becomes less rewarding and there is a shift in preference from energy-dense food rich in fat and sugar to healthier options enabling patients to adopt a favourable eating behaviour [60] (Fig. 3). These changes in eating behaviour are the result of multiple mechanisms, some of which are common to both SG and RYGB, and others that are procedure specific. These are summarised in Fig. 4.

## RYGB and SG impact on GI-derived signals

### Gut hormones

In contrast to the unfavourable gut hormone changes that accompany weight loss induced by lifestyle intervention, RYGB and SG are associated with reduced ghrelin levels and increased circulating meal-stimulated PYY and GLP-1 levels. These gut hormone changes precede and are independent from weight loss and are sustained up to 10 years post-operatively [61, 62].

### Ghrelin

Since the landmark publication by Cummings et al. [63] showing that circulating ghrelin levels rose with calorie-restricted diets but are markedly reduced post-RYGB, many studies have focused their attention on investigating the role of gut hormones as mediators of the beneficial effects of surgery. Whilst some controversy exists regarding post-RYGB-circulating ghrelin levels, these differences most likely reflect methodological variability, duration after surgery and sample processing techniques [64]. SG leads to sustained and greater reduction in circulating acyl-ghrelin levels than RYGB because of the removal of the fundus of the stomach where most ghrelin-producing cells are located [62].

### GLP-1 and PYY

Following RYGB, nutrient-stimulated circulating levels of PYY and GLP-1 are markedly elevated, most likely as a result of increased nutrient stimulation of L cells as a consequence of anatomical rearrangement. SG leads to rapid gastric emptying and enhanced exposure of L cells to nutrients with increased nutrient-stimulated PYY and GLP-1 levels, but to a lesser extent than following RYGB.

Patients with poor post-operative weight loss reported increased subjective hunger and lower satiety levels coupled with lower circulating PYY, GLP-1 and higher ghrelin, when compared with people with good weight loss [65]. These findings imply that gut hormones may play a causal role in mediating weight loss following RYGB and SG. This hypothesis is supported by three lines of evidence: first, the administration of the somatostatin analogue octreotide to people following RYGB leads to increased appetite and energy intake, and weight gain [66]; second, combined administration of di-peptidyl peptidase inhibitor (DPP4) inhibitor and exendin 9-39 (inhibiting the formation of PYY3-36 and blocking GLP-1 action) leads to increased food intake after RYGB [67]; and third profound anorexia and excessive weight loss post-SG have been shown to be associated with markedly elevated circulating fasted and post-meal PYY levels [68].

## Other gut hormones

Other gut hormones with effects on feeding behaviour have also been studied. However, the extent of their role in mediating the beneficial effects of RYGB and SG are unclear. For completeness, we have summarised these below:

Glucose-dependent insulinotropic polypeptide (GIP) is an incretin peptide hormone secreted by K-cells in the proximal small intestine. GIP increases postprandial glucagon secretion, intestinal glucose absorption and storage of fatty acids in adipose tissue [69]. Previous studies have suggested that patients with T2D are resistant to the effects of GIP and this GIP resistance has precluded the development of GIP-based T2D therapies. Following RYGB, a reduction in postprandial GIP levels has been reported [70, 71], most likely as a consequence of the K-cells being bypassed. In addition, a restoration of GIP sensitivity has also been suggested [71]. The effects of SG on circulating GIP levels have not been studied sufficiently.

Oxyntomodulin, a pro-glucagon-derived peptide with anorectic effects, is increased early after RYGB [27]. This effect has not been documented after SG.

Cholecystokinin (CCK), another anorexigenic hormone, has been suggested to act synergistically with leptin, and amylin, a pancreatic hormone co-secreted with insulin [72]. Increased levels of CCK following RYGB and SG have been reported. In one study, SG was associated with a larger CCK increase compared to the RYGB [73].

Gastrin is a peptide hormone that stimulates the secretion of gastric acid from the parietal cells of the stomach, aids in gastric motility and reduces appetite. There is some evidence suggesting that postprandial gastrin levels fall after RYGB [74] while SG may be associated with increased levels [75].

Neurotensin is co-expressed in enteroendocrine cells with GLP-1 and PYY. Circulating neurotensin levels increase after RYGB and have also been proposed to contribute to eating behaviour changes post-RYGB [76].

Additional gut hormones, such as ileal-derived FGF-19, (discussed below) may also contribute to weight loss and metabolic changes following bariatric surgery.

## Bile acids

Following RYGB and SG, changes in circulating BA levels and composition are reported and these changes are suggested to contribute to weight loss and improved glucose metabolism. Indeed, in animal models, rerouting bile to the distal small bowel by transposing the common bile duct results in improved body weight, glucose metabolism, and hepatic steatosis, and increases in plasma BA similar to those seen after RYGB. Despite their anatomical differences, RYGB and SG exert similar effects on BA, altering both their composition and circulating concentrations; however, the changes observed following SG are more modest [35, 77]. The exact mechanism responsible for elevated BA is unclear following RYGB and SG, but animal work suggests that an accelerated nutrient flow to the distal small intestine is a key mechanism [78]. The rise in circulating BA levels appears even greater several months post-operation and intestinal cellular adaptations may play a major role in explaining elevated postprandial BA levels [79]. These changes could be due to increased hepatic synthesis or altered enterohepatic recirculation of bile, or both. Metabolic procedures may also alter intestinal gut microbiota, which are key regulators of BA conjugation and secondary BA formation [80]. Post-surgery increased BA diversity might also impact on GLP-1 secretion and energy expenditure. Binding of BA TGR5 receptors in skeletal muscle and brown adipose tissue may contribute to an enhanced action of thyroid hormones by increasing energy expenditure [81]. Therefore, BA could contribute to weight loss and metabolic improvements after bariatric surgery through independent and dependent regulatory mechanisms. In RYGB subjects, bacterial overgrowth in the biliopancreatic limb may generate secondary BA species with differing affinity for FXR or TGR5 and different metabolic effects [82]. FXR gene knockout mice regained weight lost after SG, suggesting that the FXR plays a key role in mediating weight loss and metabolic improvements after SG [81]. Whether FXR signalling and/or FGF-19 contributes to the beneficial effects of bariatric surgery in humans is uncertain at present. Finally, a study measured serum BA levels before and after bariatric surgery showed that they were significantly increased only at 1-year post‐surgery; whereas, the substantial increase in PYY and GLP-1 levels could be observed as soon as 1-week post-surgery. This finding shows that increased plasma BA may not contribute to the early metabolic improvements observed after bariatric surgery [77]. Weight‐loss surgery can also affect the interplay between BA and gut microbiota, which can contribute to the metabolic effects observed in the post-operative period [83].

## Gut microbiota

Following RYGB and SG, the intestinal microbiome is altered. Animal studies with faecal transplant from RYGB-treated to germ-free mice resulted in significantly greater weight loss suggests that the altered microbiome per se contributes to weight loss [84]. Significant differences exist between the rodent and the human microbiome, and the strict relationship between microbiome and BA in humans remains to be clarified. The profound post-surgical changes in the microbiome are probably the result of anatomical, dietary and systemic changes (weight loss). In rodents, these changes can be detected as early as 7 days after RYGB [80], with similar patterns observed in humans [85]. The specific and procedure-related mechanisms responsible for post-surgery gut microbiota changes remain to be delineated [86]. In a recent study, Murphy and colleagues found that RYGB produces greater and more favourable changes in gut microbiota functional capacity than SG [87], at 1-year post-operatively, despite similar weight loss. Another study by Medina confirmed a differential effect of RYGB and SG on gut microbiota species despite equal weight loss [88]. SG seems to have fewer effects on the intestinal microbiota compared to RYGB, consistent with another study carried out in rodents [89]. This could be due to the lesser physical manipulations of the intestinal tract of SG compared to RYGB. It is difficult to conclude that gut bacteria are essential for the effects of metabolic procedures, but we can conclude that changes in gut microbiota induced by RYGB are sufficient to produce weight loss [83].

## Other mechanisms

### Enteroplasticity

Enteroplasticity refers to the post-surgical adaptations, including remodelling of the intestinal mucosa, morphologic changes, and altered innervation [35]. There is evidence that L cells proliferate following RYGB and SG and that L cells exhibit increased nutrient sensitivity, releasing more PYY and GLP-1 for a given nutrient stimulant [90].

### Glucose uptake

RYGB was recently reported to enhance SGLT1-dependent intestinal glucose uptake in the common limb and utilisation, leading to overall improvements in systemic glucose control [91]. Whether this alteration in glucose absorption is sufficient to affect whole-body glucose use, gut hormones secretion or weight loss remains uncertain [92].

### Taste and smell

Post-surgery change in appetite, taste and smell may contribute to food preference changes following RYGB and SG. Interestingly, early data suggest that RYGB and SG may differentially impact upon subjective changes in appetite, taste, olfaction and food aversion post-operatively. Large longitudinal studies combining subjective and objective measures of taste and olfaction are warranted to detect possible procedures-related effects [93].

### Vagus nerve

Afferent vagal nerve fibres in the stomach are sensitive to mechanical stretch related to food ingestion and signal to the brain [94]. Following RYGB, vagal fibres to the gastric pouch remain largely intact [95], whereas SG removes this pathway. Moreover, neurophysiological studies suggest that vagus nerve signalling also increases post-RYGB [56] and these changes may reduce food intake. These adaptations may contribute to the sustained metabolic effects of bariatric surgery.

## Conclusion

Bariatric surgery is the most effective weight loss strategy for people with severe obesity leading to reduced mortality and improvement in obesity-associated comorbidities. However, although bariatric surgery is highly effective, at the individual level, clinical response is highly variable. There are profound anatomical differences between RYGB and SG which in turn impact upon the mechanisms underlying the weight-reducing effects of these two procedures. Gut hormones, BA, gut microbiota and other mechanisms, many of them to be identified yet, contribute to the durability of decreased appetite and the sustainable weight loss following both RYGB and SG. Studies comparing weight loss suggest that after 3 years, a subtle weight loss difference may exist in favour of RYGB. Recent data suggest that longer-term health improvements are related to the degree of sustained weight loss achieved, thus highlighting the need to maximise post-surgery weight loss. A greater understanding of the procedure-related mechanisms and interaction with a person’s genetics and pre-surgery phenotype will allow surgical procedure choice to be tailored to the individual to maximise the weight loss and metabolic outcomes, and will facilitate the discovery of novel non-surgical treatments for people with obesity.

## Abbreviations

BA: Bile acids
BMI: Body mass index
CCK: Cholecystokinin
DPP4: Di-peptidyl peptidase inhibitor
FGF-19: Fibroblast growth factor-19
FXR: Farnesoid X receptor
GI: Gastrointestinal
GIP: Glucose-dependent insulinotropic polypeptide
GLP-1: Glucagon-like peptide-1
OAGB: One anastomosis gastric bypass
PYY: Peptide YY3-36
RCT: Randomised clinical trial
RYGB: Roux-en-Y gastric bypass
SG: Sleeve gastrectomy
T2D: Type 2 diabetes
